# 

**Published:** 1887-12-03

**Authors:** 


### Notice to Advertisers.

The scale of charges for small prepaid Advertisements—Situations Wanted, Situations Vacant, &c., &c., is as follows:—

| | s. | d. |
|---|---|---|
| One insertion of three lines | 1 | 0 |
| Three " " " | 2 | 6 |
| Per line afterwards | 0 | 6 |

*A line averages ten words.*

All copy for Advertisements must be sent to A. P. Watt, 2, Paternoster Square, E.C., by first post on the Tuesday *preceding* publication.

### Notice to Correspondents.

All correspondence must be written on one side of the paper only.
We cannot undertake to return MSS. not used.
**Communications,** whether intended for insertion or for private information, must be authenticated by the names and addresses of the writers, not necessarily for publication.
**Local papers** containing reports or news paragraphs **should be marked.**
It is especially requested that early intelligence of **local events of interest,** or which it may be thought desirable to bring under notice, may be sent direct to the Editor.
**All communications respecting editorial matters** should be addressed to the Editor, Norfolk House, Norfolk Street, Strand, London, W.C.

### Amusements with Profit for All Ages.

**Rules.**—All answers must be addressed to the Prize Editor, The Hospital, Norfolk House, Norfolk Street, Strand, W.C., not later than the *first* post on Thursday next. The full name and address must in all cases be given, but a *nom de plume* may be added for publication. Only one side of the paper must be written on.

No one will be permitted to win the Monthly or Quarterly Prizes twice in any one year, the annual prizes being offered especially to prevent this.

### FOR MEN AND WOMEN.

### Work Competition.

I. A PRIZE of THREE GUINEAS will be given to the competitor who sends in the best specimen of painting, which may take the form of a picture, panel, screen, flower-pots, etc., or illuminations.

II. TWO GUINEAS will be given for the best dressed doll or other piece of work suitable for presentation to the inmates of the hospitals.

III. ONE GUINEA will be given for the best scrap-book.

### Acrostic Competition.

A PRIZE of ONE GUINEA will be given to the competitor who gains the highest number of marks for best original acrostics during the ensuing quarter. All acrostics sent in for this competition will be credited according to merit, twelve marks being the highest score given for one acrostic.

A PRIZE of HALF-A-GUINEA to be given to the competitor who gains the highest score for solution of acrostics set, including the one inserted on November 26th. One mark only will be given to the competitors who solve all the lights of each acrostic.

These competitions to alternate weekly.

*Next week*, therefore, credit will be given for the *est original acrostic.*

### II.—Word and Literary Competition.

Commenced November 5th, 1887; ends January 28th, 1888.

Three prizes of one guinea, 15*s.* and 10*s.* 6*d.* will be given each quarter to the three competitors who obtain the highest number of marks: (1) by making the largest number of words derived from the word or phrase set for anatomical dissection; or (2) for the best answers to (6); or (3) by (1) and (2) combined.

(A) THE WORD COMPETITION.

We shall give the newspapers for dissection during the first quarter, that for this week being "Telegraph."

Observe.—Proper names, abbreviations, foreign words, words of less than four letters and repetitions are barred. Nuttall's *Standard* dictionary only to be used.

### Results of Third Week.

*Accepted Scores*—Ellice (19), E. E. (18), Reldas (18), Lauriston (18), Kath (16), Tim (16), Aralc (16), Brownie (15), Toby (15), Ostrich (15), Nogrub (15), Gretta (15), Condoicet (15), Puss (15), L'arc en ciel (14), Skye (12) Mater (6).

Brisbane (3,297) and Yankee (980), being new competitors, are credited this week with scores for "The Illustrated London News," which was printed in error a second time "Graphic" being the word for dissection last week All former competitors were apprised of the change.

(B) THE LITERARY COMPETITION.

For particulars of this see page 157 of last week's number. No letters have been received from across the seas this week to our great regret. Don't let our readers drag this into a precedent.

### THE CHILDREN'S CORNER.

For Prizes see page 157 of last week's issue.

### Third Monthly Competition.

### Puzzles.—First Week.

No. 1. Towns in America enigmatically expressed: 1. A man's name; the French for a town. 2. One of the eight people who went into the Ark; indisposed; a weight. 3. A globe; a nickname; a mineral. 4. A rivulet; a waterfall.

No. 2. Puzzle Sentence.—"Ave ryfi neda y."

No. 3. Riddle-me-ree.

My first is in cat, but not in dog,
My second is in peat, but not in bog,
My third is in boy, but not in man,
My fourth is in milk, but not in can,
My fifth is in one, but not in three,
My sixth is in honey, but not in bee.

No 4. Historical Charade.—My first is a part of the human body, my second is a girl's name, and my whole is a celebrated naval expedition.

Letters.—Next week give some historical facts connected with "Carisbrooke Castle."

### Results of Third Week Puzzles.

No. 9. Transpositions.

| Generals. | Their Battles. |
|---|---|
| Wellington, | Waterloo. |
| Marlborough, | Ramilies. |
| Cromwell, | Naseby. |
| Havelock, | Cawnpore. |

No. 10. Geographical Charade.—Flint Shire—Flintshire.

No. 11. Square Word

G N A T
N A M E
A M E N
T E N T

No. 12. Floral Charade—Li (lie) ly (lie)—Lily.

*All right*—A. G. S. McCulloch, Scrooge, A. C. K. Alsie, Mount Edgecumbe, Ossian, Skye, Tim, Pepita, Judy, M. S. W., The Young Canadian, Paddy, Betty Burke, Poodle, Spider. *Three right*—Fern, The Eda, Gem, W. R. Mills, Jumps.

### Holyrood Palace.

The best letters on Holyrood came naturally from Scotch children. Fern, Betty Burke, and Poodle's letters, short and to the point as they are, would be much improved by a few dates attached to the incidents. Gem's letters are always well and accurately written, and I am glad to find Skye and Ossian sending in the same good letters as formerly.

"The Eda's' Letter.

Holyrood means Holy Rood or Holy Cross, David I. founded the Abbey of the Holy Rood is gratitude for an escape from death. One day, according to tradition, he was in the forest of Drumsheugh, which is now covered over with houses and streets; whilst he was hunting, he left his followers behind him to follow after a stag who turned on him at bay and threw him down. He was just on the point of being gored to death, when a cross came miraculously into his hand and the stag fled away. So in gratitude David founded the Abbey of the Holy Cross. The Abbey was begun in 1128, and the Chapel very soon after that. The miraculous cross was placed there; the peculiar quality of this cross was, that it is said no one could make out what it was made of It is not known when the Abbey became also a Palace, but it is said Robert III. sometimes lived in Holy Rood. James I. held his court, and his little twin sons were born there in October, 1416. James II was born, crowned, and married in the Abbey of Holy Rood, and was also buried in the Chapel James IV. seems often to have lived there. Holy Rood was spelt very curiously sometimes in the old Chronicles, such as "Holy-rud hous," and "Halyrudehous." Part of the Palace that everybody knows about is Queen Mary's bedchamber; and the cabmen always point out her window as they drive people by. The bed looks very gloomy to have to sleep in and little King James VI.'s baby basket is shown. Then there is Queen Mary's dressing-room, where she sat at supper with David Rizzio, the Italian musician, of whom she was so fond. There is a stone staircase that opens into her bed room by which Earl Ruthven and the conspirators secretly approached her dressing-room on the night of the murder of her favourite; for the rude Scotch barons were jealous of her liking for a foreigner. So they rushed up these stone stairs, burst open the door, crossed her bed-room with fearful din, burst open Mary's dressing room door, and tore poor Rizzio from the Queen's presence, although she entreated and commanded them to spare him and although he clung to the skirts of his mistress's robe; dragged him from the dressing-room through the bed-room, through the presence-room into the small dark passage or landing, and there stabbed him to death. His blood, it is said, is there to-day, for the stains can never be rubbed out.

### The Tower of London.

"Gem" writes:—

The Tower of London stands on a hill, near the Thames, called Tower Hill. It was not one building, but several towers and other buildings surrounded by a wall. In olden days it was used for many purposes, viz., a citadel to defend London, a royal palace for assemblies and treaties, a state prison, a mint, an armoury, a crown treasury, and for keeping records in. The oldest parts now are of William the Conqueror; it is said to have been first used as a citadel by Julius Cæsar, but this is not likely. The gates are the Lion Gate (so-called from its being near the Royal Menagerie), the Iron Gate, the Water Gate (through which all the water business was carried on), and the Traitors' Gate, for receiving state criminals. The chief towers are: The Lion Tower, the Middle Tower, the Bell Tower (said to have been the prison of Fisher, Bishop of Rochester, and the Princess Elizabeth), the Bloody Tower (where the tradition is that Edward IV.'s two little sons were smoth red by order of Richard III., 1485), the Beauchamp Tower (the prison of Beauchamp, Earl of Warwick, 1397; and also of Anne Boleyn), the Devylin Tower, the Bowyer Tower (where the Duke of Clarence was said to have been drowned in a butt of Malmsey wine), the Brick Tower (the prison of Lady Jane Grey), the Martyrs' Tower, the Salt Tower, and the White Tower; the oldest one was founded 1078 by William the Conqueror, and finished 1098. Here took place many executions and murders, viz.: Henry VI., 1471; Sir Thomas Overbury, 1613. Queen Elizabeth's Armoury is within this tower, it has walls 14 feet thick, and includes a small cell, the prison of Sir Walter Raleigh. The Horse Armoury was built 1826, and contains a fine collection of armour. The Jewel House contains S. Edward's Crown, Queen Victoria's Crown, the Prince of Wales's coronet, and other regalia. The church of St. Peter ad Vincula is within the walls; here were buried Anne Boleyn, Catherine Howard. Lady Jane Grey, and other noted persons executed in the Tower. I have been there myself and seen it all, and I was very much interested in it. They tried to blow it up with dynamite in 1885, but did not succeed.

From "Skye."

The Tower of London is separated from the Tower Hill by a wide moat and ramparts. It is the oldest and the most historical building in England, although its exterior is very poor and mean-looking, owing to the inefficiency of the English architects at the time in which it was built. A tradition which ascribes the first building of the Tower to Julius Cæsar has been greatly assisted by Gray through the lines—

"Ye towers of Julius,
London's lasting shame,
With many a foul and midnight murder fed."

But no part of the Tower now existing is of earlier date than the Keep or White Tower, which was built by William the Conqueror in 1078. Of all English sovereigns the Tower was most enriched and adorned by Henry III., who regarded it more as a palace than a prison. In June, 1471, Henry VI. died mysteriously in the Tower after the Battle of Tewkesbury, and was said to have been stabbed by the Duke of Gloucester. Adjoining the middle gate was the Lion Tower, which was the foundation of the Zoological Gardens. It began by a present of the three leopards given to Henry III. by the Emperor Frederick. A bear was soon added to the large collection, for which the Sheriffs of London were ordered to provide a muzzle and iron chain to secure him, and a stout cord to hold him by while fishing for his dinner in the Thames. A lion and elephant were added, and it soon became quite a London sight to go and see these animals in the Tower. An arch leading to the water by a passage was called the Traitors' Gate, where poor Anne Boleyn landed, having been hurried thither without warning, and fell upon the steps praying God to defend her. At the south-west angle is the horse armoury, through which visitors are usually trotted along full speed by the warders Almost opposite Beauchamp Tower is the Tower Green—now a gravelled space, where it is said grass has never consented to grow since the execution of Hastings, 1483. A stone in the centre of the Green marks the spot on which several of the most illustrious of the tower victims have suffered death, the greater part having been executed on the Tower Hill, where they suffered the double shame of being made a show of before the mob On the Tower Green Anne Boleyn walked to her death on the scaffold, and the aged Countess of Salisbury refused to lay her head on the block, rushing round and round the scaffold, her white hair streaming in the wind, until she was hewn down by the executioner's axe. Visitors are shown over the Tower by Beefeaters, as the wardens of the Tower are called who still wear the picturesque dress of the yeomen in Henry VIII.'s time. Their name is derived from the fact that the commons of the early yeomen of the guard when on duty was beef, and the name was probably given in derision, beef being then a cheap article of food, for under Henry VIII., butchers sold mutton at three farthings and beef at only a halfpenny.

*Three Marks Each.*—Skye, Ossian, Gem, The Eda, Fern.

**In Preparation. Royal Imperial, Two Volumes, 500 Pages.**

### HOSPITALS AND ASYLUMS OF THE WORLD

THEIR HISTORY, CONSTRUCTION, ADMINISTRATION, MANAGEMENT, AND WORK.

WITH SOME 250 PLANS AND ENGRAVINGS,

**By HENRY C. BURDETT,**

*Founder of the Home Hospitals' Association for Paying Patients, and Chairman of the Executive Committee of the Hospitals Association.*

### WORKS BY THE SAME AUTHOR.

### HOSPITALS AND THE STATE: HOSPITAL MANAGEMENT AND HOSPITAL NURSING.

With Statistics of the Chief Medical Institutions in Great Britain and Ireland. [*Now Ready, Cloth* 3*s.* 6*d.*

British Medical Journal.—"The only accurate Statistics of Hospital income prepared upon an identical basis, which have ever been published."

Boston Medical and Surgical Journal.—"Mr. Burdett is one of the highest authorities in Great Britain on the subject of Hospital Administration."

### COTTAGE HOSPITALS: GENERAL, FEVER, AND CONVALESCENT.

Their Progress, Management, and Work. With an Alphabetical List of every Cottage Hospital at present opened, and Chapters on Mortuaries, Convalescent Cottages, and the Relative Mortality in Large and Small Hospitals. Second Edition now ready. [*Cloth*, 14s.

SELECTED EXTRACTS FROM REVIEWS.

Birmingham Medical Review.—"An excellent book, full of practical information."

Sanitary Record.—"We do not hesitate to say that, as a text-book of practical reference, it ought to be in the hands of everyone interested in the subject of Hospital Management, and it is gratifying to know that this opinion is shared by a very large section of the medical profession."

### PAY HOSPITALS AND PAYING WARDS THROUGHOUT THE WORLD.

Facts in support of a Re-arrangement of the English System of Medical Relief. [*Demy* 8*vo.* 6*s.*

SELECTED EXTRACTS FROM REVIEWS.

British Medical Journal.—"Mr. Burdett is well-known to the medical profession as an advocate of provident dispensaries and hospitals, and as the originator of the Home Hospitals' Association. All who are interested in these subjects will be gratified by this volume. In it the author deals with the whole question of pay hospitals and dispensaries in a large and comprehensive spirit. We commend the book to all who are interested in the improvement of medical relief—and which of us is not? It is clearly and pleasantly written, it is full of facts, and it contains many valuable suggestions. This work cannot fail to stimulate the reforming movements which have been gathering strength in this country during the last few years."

New York Medical Journal.—"Mr. Burdett has devoted many years to the study of hospital administration and management, and his book and plans are well worth perusal."

The American Practitioner.—"Mr. Burdett displays and discusses the whole scheme of hospital accommodation with a comprehensive understanding of its nature and extent, and he does it in fulness without prolixity, and in a clear catholic spirit with perspicacity. The book is a stepping-stone, a valuable contribution in the way of introduction to a review and candid reconsideration of whole subject of legal and organised charity—a theme which much demands reconsideration and readjustment in the whole civilised world. A good and timely book and suggestive."

**London: J and A. CHURCHILL, 11, NEW BURLINGTON STREET, W.**

